# Activatory Receptor NKp30 Predicts NK Cell Activation During Controlled Human Malaria Infection

**DOI:** 10.3389/fimmu.2019.02864

**Published:** 2019-12-10

**Authors:** Jona Walk, Robert W. Sauerwein

**Affiliations:** Department of Medical Microbiology and Radboud Center for Infectious Diseases, Radboud University Medical Center, Nijmegen, Netherlands

**Keywords:** malaria, *Plasmodium falciparum*, controlled human malaria infection, NK cells, NKp30, innate immunity

## Abstract

Natural killer (NK) cells are known to be activated during malaria infection, exhibiting both cytokine production and cytotoxic functions. However, NK cells are heterogeneous in their expression of surface activatory and inhibitory receptors which may influence their response to malaria parasites. Here, we studied the surface marker profile and activation dynamics of NK cells during a Controlled Human Malaria Infection in 12 healthy volunteers. Although there was significant inter-patient variability in timing and magnitude of NK cell activation, we found a consistent and strong increase in expression of the activatory receptor NKp30. Moreover, high baseline NKp30 expression was associated with NK cell activation at lower parasite densities. Our data suggest that NKp30 expression may influence the NK cell response to *P. falciparum*, explaining inter-patient heterogeneity and suggesting a functional role for this receptor in malaria.

## Introduction

Malaria infection in humans activates a broad cellular immune response involving monocytes, T cells, B cells, and NK cells. NK cells may play a functional role in protection against *Plasmodium falciparum*, as certain NK cell receptor genotypes are associated with decreases in malaria susceptibility and pathology [reviewed in (1)]. During the pathological blood stage of *P. falciparum* infection, circulating NK cells display a dual functional role, i.e., cytokine production (2–5) and killing of infected blood cells both via antibody-independent (6–8) and antibody-dependent cytotoxicity (9, 10). Their relative contribution to protection remains unknown.

NK cells are often considered a homogenous, unchanging population, but multicolored flow cytometry and mass cytometry have revealed that NK cells actually consist of many distinct populations, differing in their functionality against specific diseases (11–14). Artavanis-Tsakonas et al. previously demonstrated that in malaria naïve donors a specific subpopulation of NK cells expressing the lectin-type receptor NKG2A are the main IFN-γ producers in response to *P. falciparum*-infected RBC (15). Most studies determining the NK cell response against *P. falciparum* demonstrate that there is large inter-donor variability (16, 17). We hypothesized that this heterogeneity might at least in part be explained by differences in NK cell phenotype prior to infection.

To date most data on responsiveness of NK cells to *P. falciparum* has been obtained from *ex vivo* stimulation experiments or case-control studies in endemic areas. We took advantage of the Controlled Human Malaria Infection model to evaluate the activation and function of different NK cell subsets at multiple time points during a malaria infection. Our data show *in vivo* NK cell activation in all donors with an upregulation of IFN-γ and granzyme B production. There was indeed a significant variability both in the timing and magnitude of the NK cell response, and increased baseline receptor expression of NKp30 predicted a more rapid *in vivo* NK cell activation.

## Materials and Methods

### Clinical Trials

Study 1 was a single-center, open-label clinical trial in 12 malaria naïve individuals conducted at the Radboud university medical center (Nijmegen, The Netherlands) from May until June 2018. Study volunteers provided written informed consent and were screened as described previously (18). The trial was approved by the Central Committee on Research Involving Human Subjects (CCMO; NL63552.091.17) of the Netherlands, performed according to the Declaration of Helsinki and Good Clinical Practice and prospectively registered at ClinicalTrials.gov (NCT03454048). Volunteers were infected by the bites of five *P. falciparum* 3D7 strain-infected *Anopheles* mosquitoes, and followed up for parasitemia twice daily starting on day 6 post infection. Parasitemia was assessed by thick blood smear and qPCR. Volunteers were treated with a sub-optimal dose of piperaquine when parasitemia reached density detectable by thick blood smear or 5,000 parasites/milliliter by qPCR, and received curative treatment if recrudescent parasitemia occurred.

Study 2 was a single-center randomized placebo controlled malaria vaccine trial (CCMO NL39541.091.12; NCT01728701) published previously (19). Only study subjects that received placebo vaccination followed by CHMI were included in the current analysis. In short, volunteers received bites from five *P. falciparum* NF54 strain-infected *Anopheles* mosquitoes, and were followed up for parasitemia twice daily starting on day 5 post infection. Parasitemia was assessed by thick blood smear and/or qPCR, and volunteers received curative treatment with atovaquone/proguanil, either when parasitemia reached levels detectable by microscopy (*n* = 5) or after two consecutive qPCRs >500 parasites/milliliter (*n* = 4).

### Whole Blood NK Cell Phenotyping

In study 1, 100 μL fresh EDTA blood was stained directly with a pre-prepared and antibody mixture containing: CD3-AlexaFluor700 (Biolegend; clone OKT3), pan-γδTCR-PE (Beckman Coulter; clone IMMU510), CD56-Brilliant Violet(BV)421 (Biolegend; clone HCD56), CD16-APC-eFluor780 (eBiosciences; clone CB16), CD69-PerCP-Cy5.5 (Biolegend; clone FN50), NKp30-APC (Biolegend; clone P30-15), NKG2D-Brilliant Violet(BV)510 (Biolegend; clone 1D11), NKG2A-PEVio770 (Miltenyi Biotec; clone REA110), and CD57-FITC (Biolegend; clone HCD57). A single mixture was prepared one day before the first time point, aliquotted per time point and stored in the dark until use. Samples were stained at 4°C in the dark for 30 min, followed by erythrocyte lysis with 1 mL FACS Lysis buffer (BD Biosciences) for exactly 5 min. Samples were centrifuged and then washed with 0.5% Bovine Serum Albumin (BSA) in PBS. Cell pellets were resuspended in 100 μL 1% paraformaldehyde (PFA) and analyzed on a Gallios flow cytometer (Beckman Coulter). At each time point, staining and fixation was completed within 4 h of blood draw and flow cytometry was performed the same day using identical acquisition settings and a standardized protocol. CD69 was used as a marker for lymphocyte activation after CHMI, as described earlier (20, 21).

### PBMC Isolation and Cryopreservation

In study 2, blood samples for peripheral blood mononuclear cell (PBMC) isolation were taken pre-challenge, 3 days after antimalarial treatment and 35 days after challenge infection. Isolation and cryopreservation was performed as described previously (22). In short, PBMCs were isolated from citrate anti-coagulated blood using vacutainer cell preparation tubes (CPT; BD Diagnostics) by density gradient centrifugation. Cells were washed four times in ice-cold phosphate buffered saline (PBS), counted using 0·1% Trypan blue with 5% Zap-o-Globin II Lytic Reagent (Beckman Coulter), cryopreserved at a concentration of 10 × 10^6^ cells/ml in ice-cold fetal calf serum (Gibco)/10% DMSO (Merck), and stored in vapor-phase nitrogen.

### PBMC Thawing and Re-stimulation

Immediately prior to use, cells were thawed and washed twice in Dutch-modified RPMI 1640 (Gibco/Invitrogen). Cell viability was assessed by counting in 0·1% Trypan blue with 5% Zap-o-Globin II Lytic Reagent (Beckman Coulter) to assess cell viability. PBMCs were cultured at 2.5 × 10^6^ cells/ml in RPMI 1640 (Dutch Modification; Gibco) with 5 mg/ml gentamycin (Centraform), 100 mM pyruvate (Gibco), 200 mM glutamax (Gibco), supplemented with 10% heat-inactivated pooled human A+ serum (obtained from Sanquin Bloodbank, Nijmegen, The Netherlands) at a final volume of 200 μL in 96-wells plates. Cells were stimulated with purified *Plasmodium falciparum* NF54 schizonts or uninfected red blood cells at a concentration of 5 × 10^6^ RBC/ml. After 3 h, Brefeldin A (10 μg/mL; Sigma-Aldrich) and monansin (2 μM; eBioscience) were added to culture. After another 3 h (6 h total stimulation) cells from two stimulation replicates (1.0 × 10^6^ cells total) were combined, washed and stained with Fixable Viability Stain 700 (BD Biosciences) for 30 min. After washing with PBS, cells were stained with extracellular antibodies, CD3-AlexaFluor700 (Biolegend; clone OKT3), CD56-PE (Biolegend; clone HCD56), CD16-APC-eFluor780 (eBiosciences; clone CB16), NKG2A-PEVio770 (Miltenyi Biotec; clone REA110), and CD57-APC (Biolegend; clone HCD57) for 30 min at 4°C in the dark. Cells were washed and fixed with Foxp3 fixation/permeabilization buffer (eBioscience). After washing with permeabilization buffer (eBioscience) cells were stained for intracellular cytokines with IFN-γ-PE-Dazzle (Biolegend; clone 4S.B3) and granzyme B-FITC (Biolegend; clone GB11). After another wash with permeabilization buffer, cells from two staining replicates (2.0 × 10^6^ cells total) were taken up in 200 μL 1% paraformaldehyde (PFA) and analyzed on a Gallios flow cytometer (Beckman Coulter) the next day.

### Data Analysis and Statistics

Flow cytometry data was analyzed using Flow Jo software (version 10.0.8 for Apple OS). Statistical analysis was performed using GraphPad Prism (version 5.03 for Windows). Gating strategy and representative plots are shown in Supplementary Figure 1 (whole blood) and Supplementary Figure 3 (PBMCs).

## Results

### Heterogeneity in NK Cell Activation After CHMI

**Figure 1:**
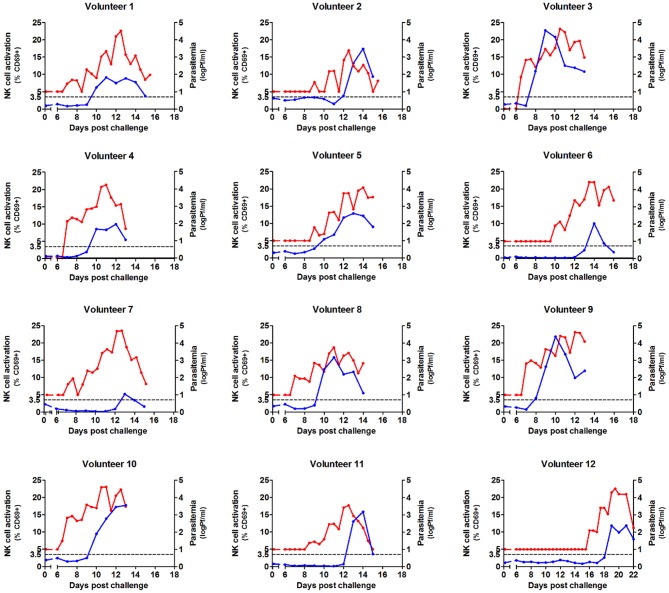
Kinetics of parasitemia and NK cell activation during Controlled Human Malaria Infection. NK cells were analyzed by flow cytometry daily in whole venous blood from 12 volunteers undergoing Controlled Human Malaria Infection. Antimalarial treatment was initiated when parasite densities reached levels detectable by microscopy. Each graph shows the activation of NK cells (defined by CD69 surface expression) from day 6 post infection until day 3 after antimalarial treatment (blue line, left axis). The same graph shows parasitemia measured by qPCR from day 6 after infection, until day 3 after antimalarial treatment (red line, right axis). NK cell activation is first seen 1–2 days after the first appearance of parasitemia. Each graph represents the data gathered for a single volunteer (*n* = 12).

After malaria infection, NK cell activation as defined by upregulated CD69 expression was determined daily from day 6 post-infection until 3 days after antimalarial treatment (Supplementary Figure 1). In study #1 the first activation of NK cells in a number of volunteers was observed 1 day after the first appearance of parasitemia detectable by qPCR (Figure 1).

**Figure 2:**
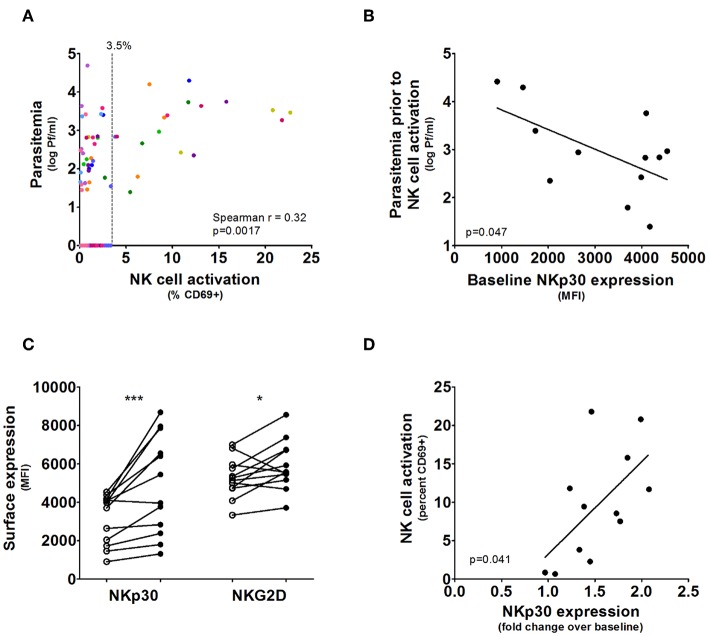
NKp30 predicts rapid NK cell activation during CHMI. **(A)** The graph shows NK cell activation correlated with parasitemia in all post-challenge but pre-treatment blood samples, where each color represents the samples from an individual volunteer. Blood samples without parasites showed up to 3.5% NK cell CD69 expression. **(B)** For each volunteer (*n* = 12) the maximum parasitemia measured by qPCR in prior to or at the moment of first NK cell activation measured in whole blood was determined. Increased baseline NK cell NKp30 surface expression determined by Mean Fluorescent Intensity correlated with NK cell activation at lower parasitemia. Line and *p*-value are the result of a linear regression analysis. **(C)** Surface expression of the activatory receptors NKp30 and NKG2D determined on total NK cells for each volunteer prior to malaria infection (open circles) and on the day of antimalarial treatment (closed circles). *P*-values are the result of Wilcoxon matched-pairs signed rank test; *p < 0.05; ***p < 0.001. **(D)** The fold change in NKp30 expression (determined by MFI) between measurements at baseline and antimalarial treatment for each volunteer were correlated to NK cell CD69 expression at on the day of antimalarial treatment. Line and *p*-value are the result of a linear regression analysis.

In the absence of parasitemia, up to 3.5% of NK cells expressed CD69, therefore >3.5% CD69 expression was considered significant NK cell activation above background (Figure 2A). There was indeed a significant heterogeneity in the timing of first NK cell activation, ranging from 1 day after the first appearance of parasitemia (i.e., volunteer 5) to 5 days after parasitemia (volunteer 7). This may be partially explained by differences in starting parasite density. Parasitemia (prior to the initiation of antimalarial treatment) correlated strongly with the degree of NK cell activation (Spearman *p* = 0.0017; Figure 2A). However, this does not explain the diversity entirely, as some volunteers have significant NK cell activation (defined as CD69 expression >3.5%) at very low circulating parasitemia, such as volunteer 5, while others require very high parasitemia before NK cells become activated, such as volunteer 7. This circulating parasite density prior to NK cell activation was highly variable between volunteers (mean 4,798 Pf/ml, range 25–26,152 Pf/ml), suggestive for a host-dependent factor.

### Baseline NKp30 Expression Predicts Activation After CHMI

NK cell activation is dependent on a delicate balance between activatory- and inhibitory receptors, and the expressed receptor profile may relate to the observed heterogeneity during CHMI. Therefore, we next determined whether the expression of activatory receptors NKp30 or NKG2D, the inhibitory receptor NKG2A or the differentiation marker CD57 predicted an individual's response to CHMI. Indeed, higher baseline NK cell NKp30 expression correlated with activation at lower parasitemia (linear regression *p* = 0.047; Figure 2B). NKp30 and NKG2D were expressed on nearly 100% of NK cells for all volunteers (Supplementary Figure 1).

NKp30 was strongly upregulated during CHMI (pre-challenge vs. day of antimalarial treatment: mean MFI 3,145 vs. 4,913, Wilcoxon matched-pairs signed rank test *p* = 0.0010; Figure 2C), while the upregulation of NKG2D was marginal (pre-challenge vs. day of antimalarial treatment: mean MFI 5,268 vs. 5,916, Wilcoxon matched-pairs signed rank test *p* = 0.043; Figure 2C). The increase in NKp30 expression was proportional to total NK cell activation at antimalarial treatment (linear regression *p* = 0.041; Figure 2D).

**Figure 3:**
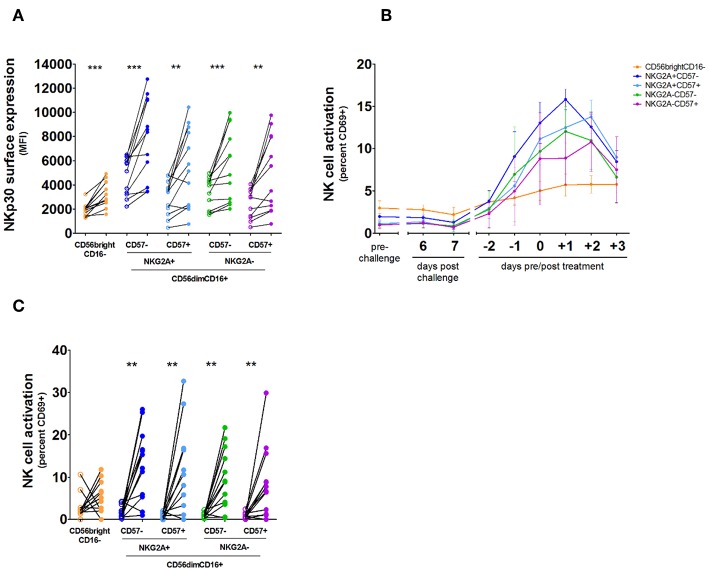
NKp30 expression and activation kinetics on NK cell subsets. NK cells were analyzed by daily flow cytometry in whole venous blood from 12 volunteers undergoing Controlled Human Malaria Infection. Antimalarial treatment was initiated when parasite densities reached levels detectable by microscopy. **(A)** Total NK cells were divided into five subpopulations based on their surface expression of CD56, CD16, NKG2A, and CD57: CD56^bright^CD16– (orange), CD56^dim^CD16+NKG2A+CD57– (dark blue), CD56^dim^CD16+NKG2A+CD57+ (light blue), CD56^dim^CD16+NKG2A-CD57– (green), and CD56^dim^CD16+NKG2A-CD57+ (purple). Surface expression of NKp30 for each NK cell subset prior to malaria infection (open circles) compared with NKp30 expression on each NK cell subset on the day of antimalarial treatment (closed circles). *P*-values are the result of Wilcoxon matched-pairs signed rank test; **p < 0.01; ***p < 0.001. **(B)** The graph shows the mean and error of NK cell CD69 surface expression on each subset per day in 12 volunteers. **(C)** Surface expression of CD69 was determined for each NK cell subset prior to malaria infection (open circles) and on the day of antimalarial treatment (closed circles). *P*-values are the result of Wilcoxon matched-pairs signed rank test; *p < 0.05; **p < 0.01; ***p < 0.001.

NK cells can be divided into distinct populations representing levels of differentiation based on their expression of CD56, CD16, NKG2A, and CD57 (11), and a previous study suggested NKG2A+ NK cells are more responsive to *P. falciparum in vitro* (15). We sought to determine whether this may result from differential expression of NKp30. However, while baseline expression of NKp30 varied between CD56dimNKG2A+ and CD56dimNKG2A– subsets (Figure 3A; Supplementary Figure 2), all NK cell subsets showed an upregulation of NKp30 (Figure 3A). Furthermore, we did not see any differences in activation as defined by CD69 upregulation between the CD56dim subsets, though there was significantly more activation of the CD56dim subset compared to the CD56bright subset (Figures 3B,C).

### NK Cell Subsets Upregulate CD69, IFN-γ, and Granzyme B During CHMI

**Figure 4:**
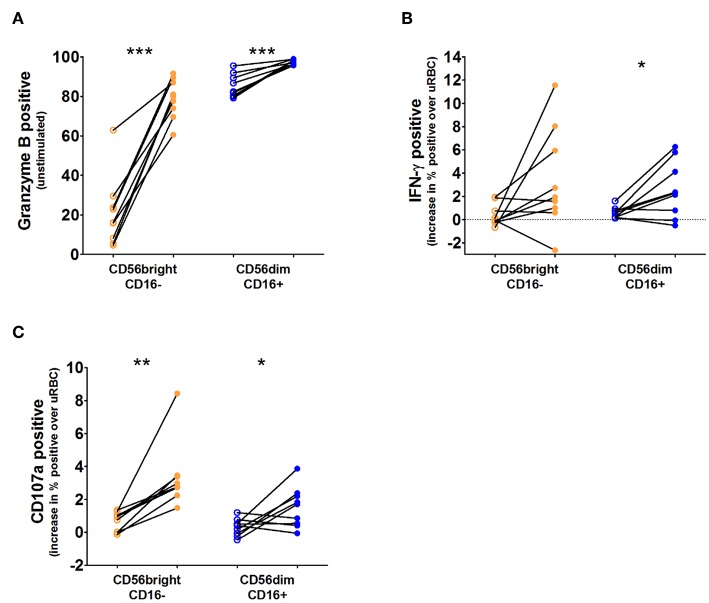
IFN-γ, granzyme B, and degranulation responses per NK cell subset. Cryopreserved PBMCs from nine volunteers taken before challenge (open circles) and 3 days after antimalarial treatment (closed circles) were thawed and stimulated for 6 h with *Pf*-infected red blood cells (PfRBC) or uninfected RBC (uRBC). Total NK cells were divided into two subpopulations based on their surface expression of CD56 and CD16. **(A)** Intracellular granzyme B content (% cells positive) determined by flow cytometry in unstimulated NK cells (those incubated with uninfected RBC cultures) at both time points. **(B)** Intracellular IFN-γ production (% cells positive) in response to PfRBC stimulation at both time points, IFN-γ production after PfRBC stimulation was corrected for production in response to uRBC. **(C)** CD107a staining (% cells positive) in response to PfRBC stimulation at both time points, CD107a staining after PfRBC stimulation was corrected for production in response to uRBC. For all three graphs open circles are pre-infection time points and closed circles are day 3 post antimalarial treatment. *P*-values are the result of paired samples *t*-test; *p < 0.05; **p < 0.01; ***p < 0.001.

As there appears to be little activation of the CD56bright NK cell subset during the course of infection, we wanted to determine the ability of both the CD56brightCD16– and CD56dimCD16+ subsets to produce granzyme B and IFN-γ and degranulate during infection, using isolated and cryopreserved peripheral blood mononuclear cells (PBMCs) from study #2 (Supplementary Figure 3). We found that both subsets increase production of granzyme B and IFN-γ and show improved degranulation during infection (Figures 4A–C; Supplementary Figure 4).

## Discussion

These data show that NKp30 is a marker for the NK cell response during a Controlled Human Malaria Infection, and suggests a possible functional role in the response to infected red blood cells. We demonstrate that the expression of this receptor at baseline relates to individual NK cell responses to *P. falciparum in vivo*. Furthermore, we show that NK cell activation during the course of infection is linked to an increase in NKp30 expression.

Both NKp30 and NKG2D have been shown to increase expression during NK cell activation (14, 23), however, during CHMI the magnitude of NKp30 upregulation of is particularly pronounced compared to NKG2D. It has previously been demonstrated *in vitro* that NKp30 binds to the *P. falciparum* protein *Pf* EMP1 leading to NK cell activation (24). This supports our finding that NK cells with higher resting NKp30 expression are more sensitive to activation at lower parasitemia. However, it is important to note that other *in vitro* studies suggest that *Pf* EMP1 may be dispensable (25) and that MDA5 signaling may be essential (26) for NK cell activation in response to *Pf* RBC. Therefore, multiple mechanisms may be involved in NK cell activation during malaria.

This is the first study with longitudinal daily samples from the initial phase of a malaria infection as parasites emerge from the liver that suggests an important role for NKp30. We thereby measured CD69 expression directly in patient blood samples, without re-stimulation, remaining close to the induced *in vivo* phenotype of an early natural infection. Furthermore, we show that baseline NKp30 expression is linked to a more rapid NK cell activation during subsequent infection.

Population based studies conducted in sub-Saharan Africa have identified a single nucleotide polymorphism (SNP) in the promoter for the NCR3 gene that encodes NKp30 that is associated with an increased number of clinical, uncomplicated malaria episodes in individuals over 5 years old (27–29). The combined data are highly suggestive for a potential functional role of NKp30-mediated NK cells in malaria. In our study we do not detect differences in time to parasitemia, maximum parasitemia, or parasite multiplication rate between those with high NKp30 expression and those with low NKp30 expression (data not shown). However, an important limitation of this study is that it was not designed to measure an effect on control of blood stage parasite replication. Even in volunteers with very rapid NK cell activation, this occurred only 2 days before the initiation of antimalarial treatment. This period between NK cell activation and drug treatment would be too short to measure an effect on parasite multiplication. Instead, cohort studies in endemic areas are better suited to answer this question. Future studies in endemic areas could determine NKp30 expression on NK cells at the beginning of a malaria season and during follow-up visits, and correlate this with number of clinical malaria episodes.

Broad inter-donor variability in the activation of NK cells in response to *P. falciparum* has been described in multiple studies (16, 17, 21). Our current finding suggest that baseline NK cell phenotype can play a role in this diversity. However, other immunological factors, including other activatory and inhibitory receptors not studied here, interactions with other immune cells and cytokine production likely also contribute to the NK cell response. Furthermore, parasitological factors, such as the initial starting parasitemia and parasite multiplication rate may also affect host response.

The phenotypic diversity of NK cells has been a topic of extensive study during the last decade (12, 14). Since the first discovery of NK cell memory in murine CMV infection (30), specific NK cell phenotypes have been identified as the main responders in human EBV (13), CMV (31), and HIV infection (14, 32) as well. Similarly, studies suggested that NKG2A+ NK cells, specifically respond to *P. falciparum* (15, 16). Interestingly, this does not appear to be the case during controlled human malaria infection *in vivo*.

Nevertheless, the finding that NKp30 expression predicts the response to CHMI, underscores the potential importance of NK cell phenotype in our susceptibility to disease. The diversity of the NK cell repertoire has been implicated in the risk of HIV acquisition (14, 33), and viral infections in turn have been shown to change its composition (34–37). Our study suggests that NK cell phenotype affects the response to a *P. falciparum* infection.

The current study was limited to analysis of CD56, CD16, NKG2A, CD57, NKp30, and NKG2D. In contrast, data from studies on other diseases using cytometry by time-of-flight (CyTOF) have suggested there may be more than 100,000 NK cell phenotypes, each characterized by a distinct combination of surface receptors (12). Furthermore, the expression of diverse killer cell immunoglobulin-like receptors (KIRs) plays an important role in NK differentiation and function (11). Therefore, it is likely that additional receptors, or combinations of inhibitory and activatory receptors, are also important for the interaction between NK cells and *P. falciparum* parasites. Future studies looking at a larger number of receptors and cytokines could unravel both these effects in more detail.

In conclusion, this study is the first to identify the expression the NK cell activatory receptor NKp30 as a marker that predicts a rapid NK cell response to parasitemia and suggest a potential role for this receptor in NK cell functionality against *P. falciparum*.

## Data Availability Statement

The datasets generated for this study are available on request to the corresponding author.

## Ethics Statement

The studies involving human participants were reviewed and approved by the Central Committee on Research Involving Human Subjects, Netherlands. The patients/participants provided their written informed consent to participate in this study.

## Author Contributions

JW and RS designed the study. JW performed the analysis and wrote the first draft of the manuscript which was supervised by RS.

### Conflict of Interest

The authors declare that the research was conducted in the absence of any commercial or financial relationships that could be construed as a potential conflict of interest.
