# Supplementary material for: Activatory Receptor NKp30 Predicts NK Cell Activation During Controlled Human Malaria Infection
Source: Front Immunol. 2019 Dec 10;10:2864. doi: 10.3389/fimmu.2019.02864 (PMC6916516; doi:10.3389/fimmu.2019.02864)
Supplement: Supplementary file 1 [file Data_Sheet_1.docx]

**Supporting Information**

**Supplementary figure 1: Gating strategy whole blood NK cell flow cytometry.** Lymphocytes were gated from fresh whole blood based on forward- and side-scatter characteristics. Duplets were excluded from the analysis. NK cells were identified as CD3-CD56+ cells, and further divided into CD56dimCD16+ and CD56brightCD16-. CD56dimCD16+ NK cells were further split into four populations based on NKG2A and CD57 expression. In all samples NKp30 was expressed on nearly all NK cells and quantified based on mean fluorescent intensity (MFI). CD69 was expressed on a subset of cells, only during parasitemia, and quantified as percent positive cells.

**
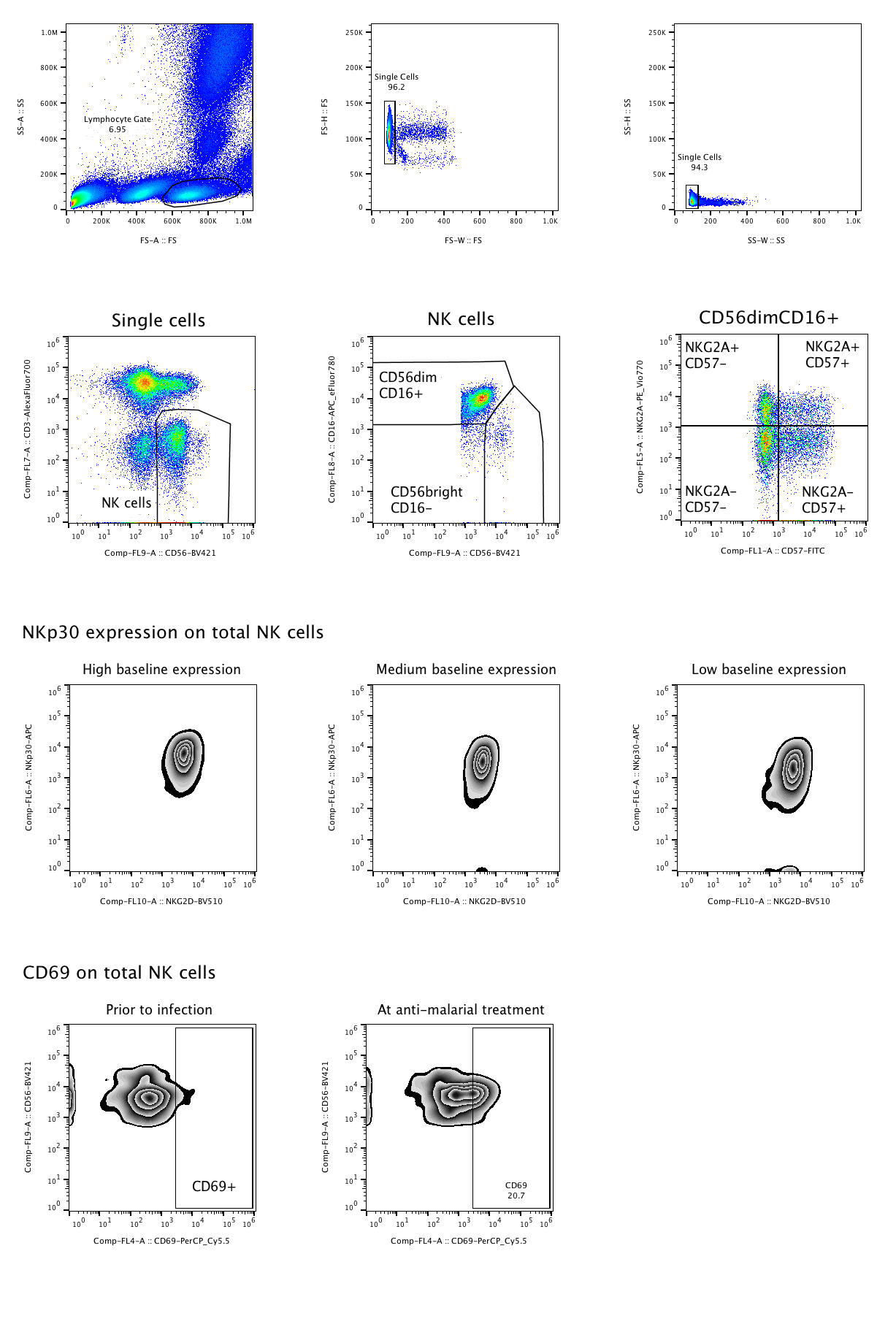
**

**Supplementary figure 2: NKp30 expression on NK cell subsets at baseline.** NK cells were analyzed by daily flow cytometry in whole venous blood from 12 volunteers undergoing Controlled Human Malaria Infection. Antimalarial treatment was initiated when parasite densities reached levels detectable by microscopy. Total NK cells were divided into five subpopulations based on their surface expression of CD56, CD16, NKG2A and CD57: CD56brightCD16- (orange), CD56dimCD16+NKG2A+CD57- (dark blue), CD56dimCD16+NKG2A+CD57+ (light blue), CD56dimCD16+NKG2A-CD57- (green), and CD56dimCD16+NKG2A-CD57+ (purple). Surface expression of NKp30 was determined for each NK cell subset prior to malaria infection at baseline. P-values are the result of Friedman test with Dunn’s multiple comparison test; * p<0.05; **p<0.01; ***p<0.001.

**
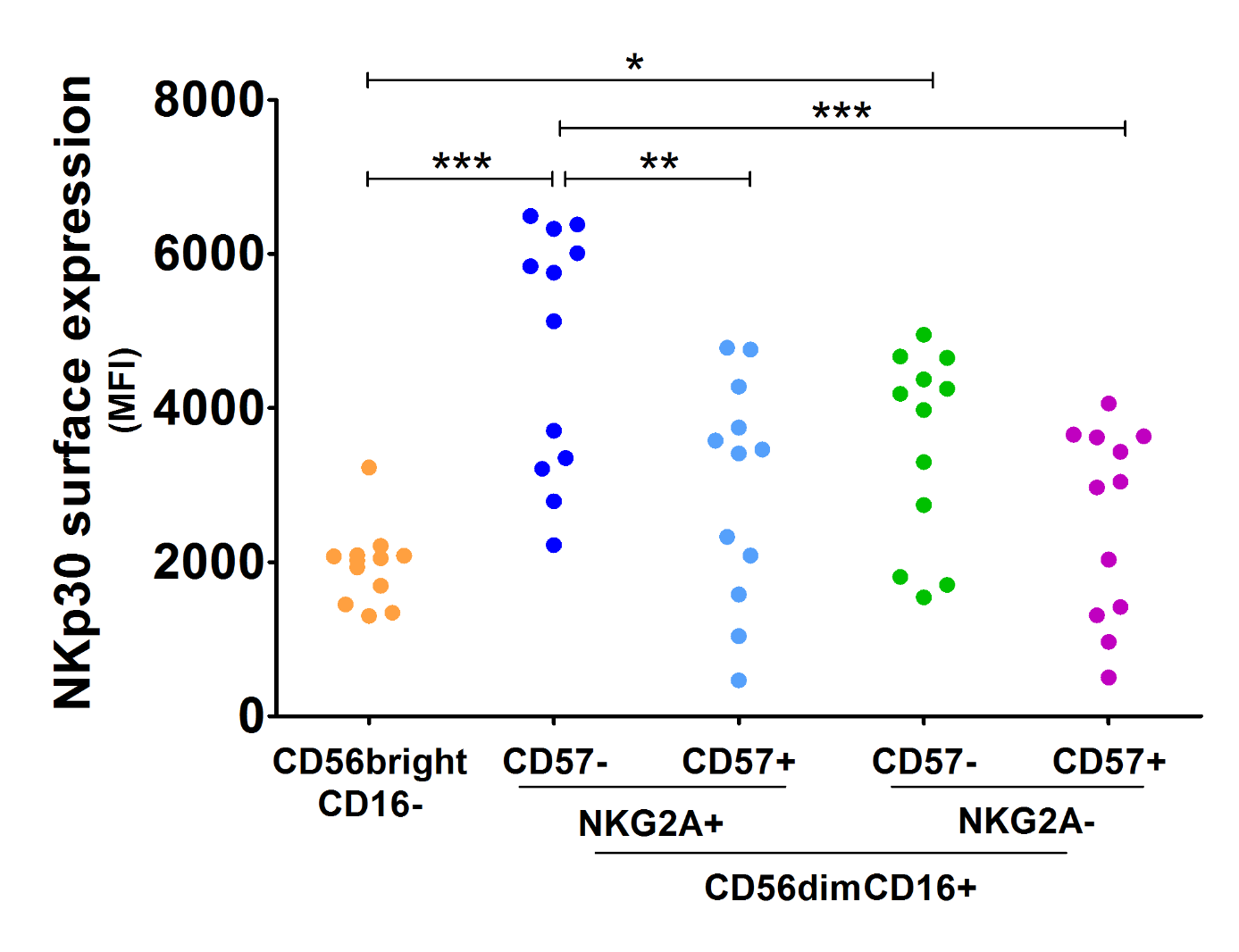
**

**Supplementary figure 3: Gating strategy PBMC stimulation flow cytometry.** Lymphocytes were gated from cryopreserved PBMCs based on forward- and side-scatter characteristics. Duplets were excluded from the analysis. Viability dye negative, CD3- cells were selected. From live CD3- cells NK cells were identified as either CD56dimCD16+ and CD56brightCD16-. Granzyme B was expressed on a subset of NK cells at all timepoints. The gate was defined using total live CD3- cells, as they show a clear negative and positive population, examples of this gate, and NK cell populations with a high- and low number of granzyme B positive cells are shown. IFN-γ and CD107a were expressed on a subset of cells only after stimulation and quantified as percent positive cells.

**
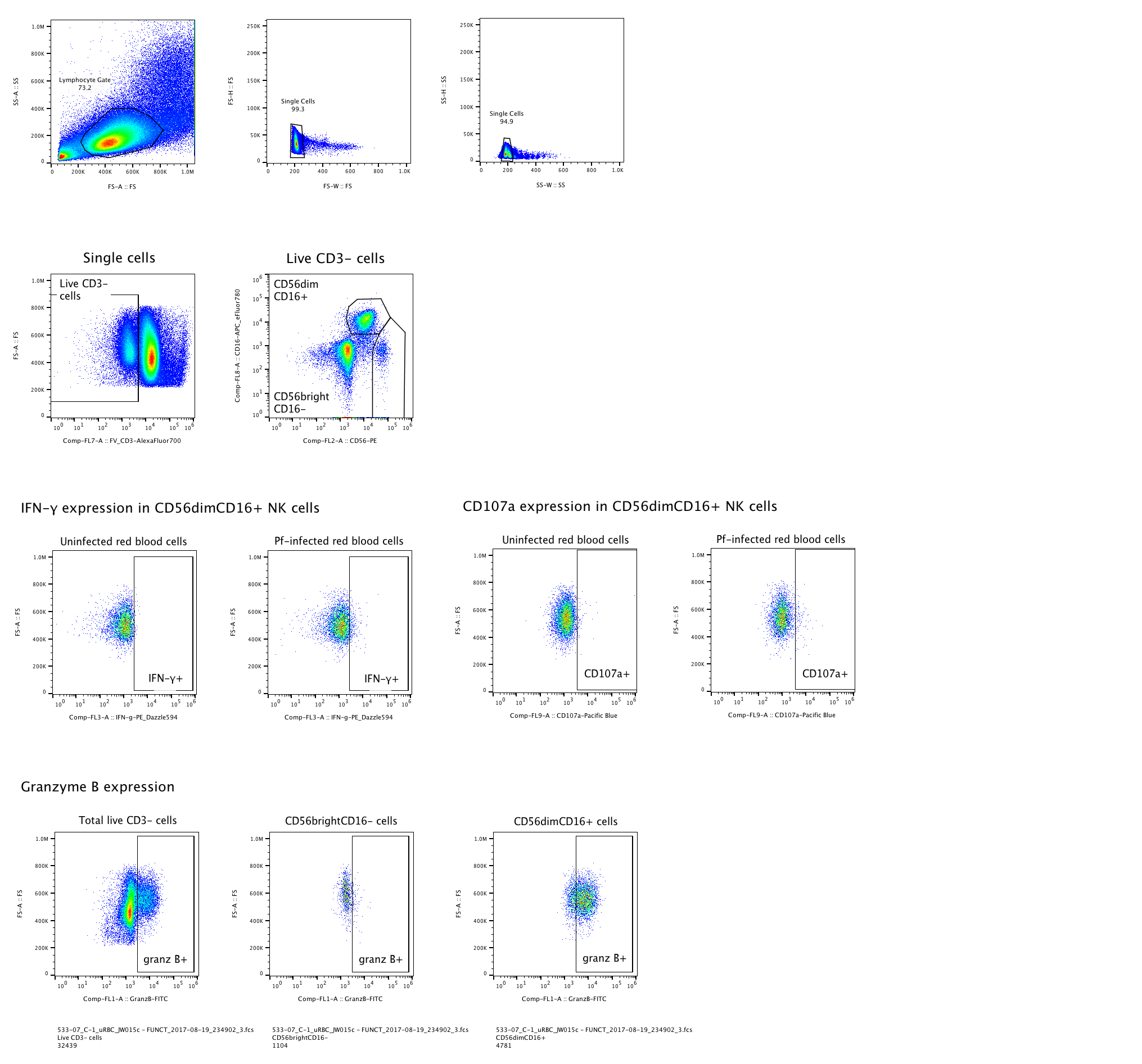
**

**Supplementary figure 4: IFN-γ production and degranulation in response to *P. falciparum.*** Cryopreserved PBMCs from 9 volunteers taken before challenge and three days after antimalarial treatment were thawed and stimulated for 6 hours with *Pf*-infected red blood cells (PfRBC) or uninfected RBC (uRBC). Total NK cells were divided into two subpopulations based on their surface expression of CD56 and CD16. **(A)** Intracellular IFN-γ production (% cells positive) in response to PfRBC and uRBC stimulation at both time points **(B)** Degranulation defined as CD107a staining (% cells positive) after PfRBC and uRBC stimulation at both time points.

**
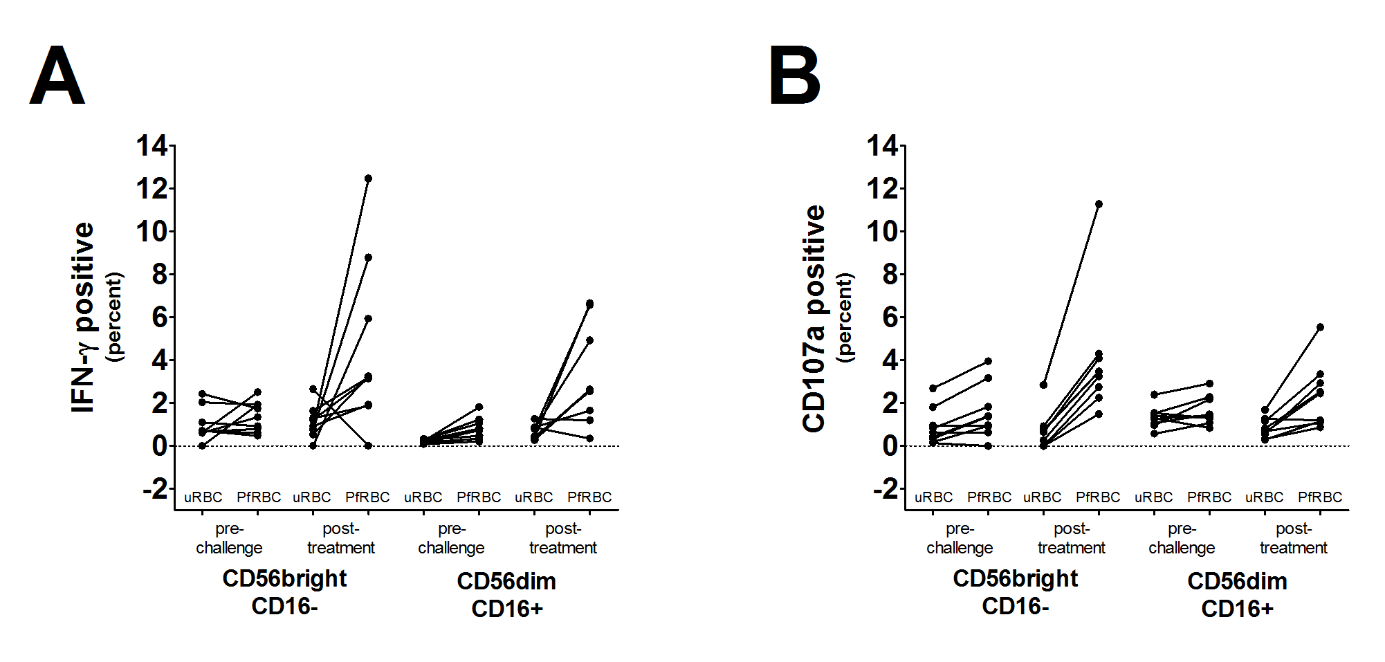
**
